# A Coarse Alignment Algorithm Based on Vector Reconstruction via Sage–Husa AKF for SINS on a Swaying Base

**DOI:** 10.3390/s25175274

**Published:** 2025-08-25

**Authors:** Yongyun Zhu, Bingbo Cui, Dianlei Han, Yaohui Zhu, Yuanyuan Gao, Shede Liu

**Affiliations:** 1 School of Agricultural Engineering, Jiangsu University, Zhenjiang 212013, China; yongyunzhu@ujs.edu.cn (Y.Z.); cuibingbo@ujs.edu.cn (B.C.); handianlei@ujs.edu.cn (D.H.); yaohui_zhu@ujs.edu.cn (Y.Z.); 2 Key Laboratory of Modern Agricultural Equipment and Technology, Ministry of Education, Zhenjiang 212013, China; 3 School of Instrument Science & Engineering, Southeast University, Nanjing 210096, China; 230208374@seu.edu.cn

**Keywords:** coarse alignment, vector reconstruction, Sage–Husa, adaptive Kalman filter, SINS

## Abstract

As a rapid self-alignment method, the coarse alignment technique, under swaying-base conditions, can enhance initial attitude determination speed without external aids, which is critical for strapdown inertial navigation systems (SINSs). The inaccuracy of the observation vector model caused by inertial sensor error accumulation and external disturbances is a critical constraint on the performance of coarse alignment methods. To address the above issues, a coarse alignment algorithm based on vector reconstruction via the Sage–Husa adaptive Kalman filter (AKF) is proposed in this paper. First, an apparent velocity vector observation model was established. Second, a sliding-window vector integration algorithm was designed to process this observation model, aiming to reduce the cumulative error of the observation vector. Next, a vector reconstruction model based on the Sage–Husa AKF algorithm was designed, and the self-alignment process was completed using the reconstructed observation vector. Finally, simulations and turntable experiments were conducted to demonstrate the effectiveness of the method. The results indicate that this method exhibits alignment performance superior to that of similar coarse alignment methods.

## 1. Introduction

The autonomous driving and operation of Autonomous Mobile Systems (AMSs) are crucial to improving operational efficiency, addressing labor shortages, and enhancing safety in high-risk operations [1,2,3,4], constituting a major research trend across industrial and agricultural domains. Positioning and attitude determination constitute the fundamental technical basis for AMS to achieve environmental perception, path planning, and precise operation [5,6,7,8]. Multiple viable positioning methods exist for AMSs, primarily including Global Navigation Satellite Systems (GNSSs) [9,10], Strapdown Inertial Navigation Systems (SINSs) [11,12], and Vision Navigation [13,14]. However, achieving high-precision and high-update-rate attitude detection primarily relies on SINS. With its strong autonomy, high update frequency, and excellent dynamic performance, an SINS can provide accurate attitude determination for AMSs [15,16,17].

By processing the raw measurement data acquired from inertial measurement units (IMUs), an SINS computes the relative motion state variation of the carrier with respect to its initial reference state [18]. As a dead-reckoning navigation system, an SINS requires critically initialized parameters comprising attitude, velocity, and position to guarantee subsequent navigation precision. The initial velocity and position of the carrier can be obtained by sensors such as a GNSS or a priori information, while the acquisition of initial attitude requires the design of dedicated initial alignment algorithms [19,20]. Alignment speed and alignment accuracy are two critical performance metrics for the initial alignment algorithms [21,22].

Chronologically, the initial alignment process can be divided into coarse and fine alignment and stages [21,23]. The attitude solved by the coarse alignment algorithm serves as the fine alignment algorithm’s initial condition. Fine alignment typically employs data fusion methods, such as a Kalman filter, to estimate inertial sensor errors, yielding a high-precision attitude solution [24,25]. Therefore, the primary objective of coarse alignment is to rapidly converge the attitude error within a small-angle error range to enhance the convergence speed of the initial alignment process [20,26]. In practical applications, coarse alignment under static-base conditions exhibits inherent limitations. Hence, swaying-base coarse alignment currently represents the most efficient approach. This paper is primarily directed at the design of a rapid coarse alignment algorithm for a swaying base.

Extensive research on coarse alignment methods has been conducted by scholars worldwide [20,22,26,27]. Among these endeavors, the inertial frame coarse alignment method proposed by Yan Gongmin stands as a representative approach [27]. This method employs a decomposition of the body attitude matrix into a product of several sub-matrices and achieves rapid initial attitude determination based on the dual-vector attitude determination principle. Subsequently, some scholars have made improvements based on this method [28]. However, in general, coarse alignment methods utilizing dual-vector attitude determination do not fully exploit the vector information throughout the entire alignment process, resulting in limited alignment performance. Building upon the inertial frame coarse alignment method, Wu Yuanxin [29] proposed an optimization-based alignment (OBA) method, reformulating the coarse alignment problem as an attitude determination problem of a constant attitude matrix. Subsequently, scholars have successively incorporated attitude determination methods such as q-method, Quaternion Estimator (QUEST), and Recursive QUEST (REQUEST) into coarse alignment methods to enhance alignment performance [30,31,32]. Xu Xiang [33,34] et al. argued that in the coarse alignment observation vector model, the observation vectors contain accumulated errors due to inertial sensor inaccuracies, which constitute the primary factor limiting convergence speed. Therefore, Xu [34] conducted research on Kalman filter-based vector reconstruction algorithms. Inspired by the work of Xu Xiang et al., this paper incorporates an investigation of a vector reconstruction method based on the Sage–Husa adaptive Kalman filter (AKF) algorithm for application in coarse alignment algorithms to accelerate convergence speed.

The remainder of this paper is organized as follows. The vector observation model for coarse alignment is devoted in Section 2. The vector reconstruction model is constructed in Section 3. The effectiveness and practicability of the proposed algorithm is demonstrated through simulation and turntable tests in Section 4 and Section 5. Finally, conclusions are drawn in Section 6.

## 2. The Vector Observation Model for Coarse Alignment

In this section, an apparent velocity vector observation model based on sliding-window vector integration is constructed, utilizing the principle of coarse alignment in the inertial frame.

### 2.1. Definition of Coordinate Systems

The coordinate systems used in the paper are defined as follows:

(1) *i*-frame: inertial frame, Earth-centered initially fixed, orthogonal reference frame.
(2) *n*-frame: navigation frame, orthogonal reference frame aligned with East–North–Up (ENU) geodetic axes.
(3) *n*0-frame: orthogonal reference frame non-rotating relative to the *i*-frame, forming the *n*-frame at the start-up in the inertial space.
(4) *b*-frame: body frame, Right–Forth–Up orthogonal frame aligned with IMU axes.
(5) *b*0-frame: orthogonal reference frame non-rotating relative to the *i*-frame, forming the *b*-frame at the start-up in the inertial space.
(6) *e*-frame: Earth frame, Earth-centered Earth-fixed (ECEF) orthogonal reference frame.
(7) *e*0-frame: orthogonal reference frame non-rotating relative to the *i*-frame, forming the *e*-frame at start-up in the inertial space.

### 2.2. Decomposition of Attitude Matrix

The attitude matrix $C_{b}^{n}\left(t\right)$ at any time *t* can be decomposed via the chain rule:

(1) $$C_{b}^{n}\left(t\right)=C_{b\left(t\right)}^{n\left(t\right)}=C_{n0}^{n\left(t\right)}C_{b0}^{n0}C_{b\left(t\right)}^{b0}$$

where $C_{b0}^{n0}$ is the attitude matrix corresponding to the initial moment. The matrices $C_{n0}^{n\left(t\right)}$ and $C_{b\left(t\right)}^{b0}$ are the attitude change matrices of the *n*-frame and *b*-frame, respectively, from time $t_{0}$ to *t*. Obviously, the initial values of the attitude matrices $C_{n0}^{n\left(t\right)}$ and $C_{b\left(t\right)}^{b0}$ are both identity matrices, i.e., $C_{n0}^{n\left(t_{0}\right)}=C_{b\left(t_{0}\right)}^{b0}=I_{3}$. The differential equations of the matrices $C_{n0}^{n\left(t\right)}$ and $C_{b\left(t\right)}^{b0}$ are expressed as

(2) $${\dot{C}}_{n\left(t\right)}^{n0}=C_{n\left(t\right)}^{n0}\left[\omega_{in}^{n}\times \right]$$

(3) $${\dot{C}}_{b\left(t\right)}^{b0}=C_{b\left(t\right)}^{b0}\left[\omega_{ib}^{b}\times \right]$$

where the matrix $C_{n\left(t\right)}^{n0}$ is the transpose matrix of original matrix $C_{n\left(t\right)}^{n0}$, namely $C_{n\left(t\right)}^{n0}={\left(C_{n0}^{n\left(t\right)}\right)}^{T}$. The matrix ${\dot{C}}_{n\left(t\right)}^{n0}$ is the derivative of matrix $C_{n\left(t\right)}^{n0}$. The angular velocity $\omega_{in}^{n}$ is the projection vector of the rotational angular velocity of the *n* -frame relative to the *i*-frame in the *n*-frame, which can be calculated from the Earth’s rotation rate and geographical information. The matrix $\left[\omega_{in}^{n}\times \right]$ represents the skew-symmetric matrix of vector $\omega_{in}^{n}$. The matrix ${\dot{C}}_{b\left(t\right)}^{b0}$ is the derivative of matrix $C_{b\left(t\right)}^{b0}$. The angular velocity $\omega_{ib}^{b}$ is the projection vector of the rotational angular velocity of the *b*-frame relative to the *i*-frame in the *b*-frame, which can be measured by gyroscopes. The matrix $\left[\omega_{ib}^{b}\times \right]$ represents the skew-symmetric matrix of vector $\omega_{ib}^{b}$.

### 2.3. Apparent Gravity Vector Observation Model

When the vehicle is under a swaying base, the linear velocity of the vehicle is zero. Then, the specific force equation can be simplified as

(4) $$0=C_{b}^{n}\left(t\right)f^{b}+g^{n}$$

where 0 denotes a three-dimensional null vector. $f^{b}$ denotes the specific force vector measured by the accelerometer. $g^{n}$ denotes the gravitational acceleration vector.

Combining Equations (1) and (4) gives the following:

(5) $$C_{n0}^{n\left(t\right)}C_{b0}^{n0}C_{b\left(t\right)}^{b0}f^{b}=-g^{n}$$

Multiplying both sides of the above equation by the matrix $C_{n0}^{b0}C_{n\left(t\right)}^{n0}$ yields the following:

(6) $$C_{b\left(t\right)}^{b0}f^{b}=-C_{n0}^{b0}C_{n\left(t\right)}^{n0}g^{n}$$

Through Equation (6), the vector observation model based on the apparent gravity can be obtained:

(7) $$\beta_{a}=-C_{n0}^{b0}\alpha_{a}$$

where

(8) $$\left\{\begin{array}{l} \beta_{a}=f^{b0}=C_{b\left(t\right)}^{b0}f^{b}\\ \alpha_{a}=g^{n0}=C_{n\left(t\right)}^{n0}g^{n} \end{array}\right.$$

The apparent gravity vector observation model presented above is derived based on the ideal specific force equation. As shown in Equation (8), the reference vector $g^{n0}$ is calculated by transforming the gravitational acceleration vector from the *n*-frame to the *n*0-frame. Since the vehicle’s geographic position is known and fixed, the reference vector $g^{n0}$ can be considered error-free. Conversely, the observation vector $f^{b0}$ is computed by transforming the specific force vector from the *b*-frame to the *b*0-frame. In an actual SINS, measurement errors in the data provided by the IMU must be considered. Let the error-containing outputs of the gyroscope and accelerometer be denoted as ${\tilde{\omega }}_{ib}^{b}$ and ${\tilde{f}}^{b}$, respectively, and their expressions are as follows:

(9) $${\tilde{\omega }}_{ib}^{b}=\omega_{ib}^{b}+\varepsilon^{b}+w_{g}^{b}$$

(10) $${\tilde{f}}^{b}=f^{b}+\nabla^{b}+w_{a}^{b}$$

where $\varepsilon^{b}$ represents the gyroscope bias vector, $w_{g}^{b}$ represents the gyroscope measurement noise vector, $\nabla^{b}$ represents the accelerometer bias vector, and $w_{a}^{b}$ represents the accelerometer measurement noise vector.

Since the gyroscope measurements contain errors, the matrix $C_{b\left(t\right)}^{b0}$ computed via Equation (3) accumulates these inaccuracies. Consequently, Equation (3) is reformulated to express the practically computed matrix as

(11) $${\dot{\tilde{C}}}_{b\left(t\right)}^{b0}={\tilde{C}}_{b\left(t\right)}^{b0}\left[{\tilde{\omega }}_{ib}^{b}\times \right]$$

where, the matrix ${\tilde{C}}_{b\left(t\right)}^{b0}$ is an expression of matrix $C_{b\left(t\right)}^{b0}$ with an error component. The matrix ${\dot{\tilde{C}}}_{b\left(t\right)}^{b0}$ is the derivative of matrix ${\tilde{C}}_{b\left(t\right)}^{b0}$.

The matrix ${\tilde{C}}_{b\left(t\right)}^{b0}$ can be expressed as the product of its true value $C_{b\left(t\right)}^{b0}$ and the attitude error matrix between them:

(12) $${\tilde{C}}_{b\left(t\right)}^{b0}=\left[I_{3}-\left({\tilde{\phi }}^{b0}\times \right)\right]C_{b\left(t\right)}^{b0}$$

where ${\tilde{\phi }}^{b0}$ denotes the misalignment angle between matrices ${\tilde{C}}_{b\left(t\right)}^{b0}$ and $C_{b\left(t\right)}^{b0}$. On one hand, ${\tilde{\phi }}^{b0}$ depends on the magnitude of gyro bias $\varepsilon^{b}$. On the other hand, it grows progressively with time.

Substituting Equation (10) into Equation (8) and ignoring second-order terms yields the following:

(13) $$\begin{array}{ccc} f^{b0} & = & C_{b\left(t\right)}^{b0}f^{b}=\left[I_{3}+\left({\tilde{\phi }}^{b0}\times \right)\right]{\tilde{C}}_{b\left(t\right)}^{b0}\left({\tilde{f}}^{b}-\nabla^{b}-w_{a}^{b}\right)\\ & \approx & {\tilde{C}}_{b\left(t\right)}^{b0}{\tilde{f}}^{b}+\left({\tilde{\phi }}^{b0}\times \right){\tilde{C}}_{b\left(t\right)}^{b0}{\tilde{f}}^{b}-{\tilde{C}}_{b\left(t\right)}^{b0}\left(\nabla^{b}+w_{a}^{b}\right) \end{array}$$

Substituting Equation (13) into Equation (7) and rearranging terms yields the following:

(14) $${\tilde{C}}_{b\left(t\right)}^{b0}{\tilde{f}}^{b}+\left({\tilde{\phi }}^{b0}\times \right){\tilde{C}}_{b\left(t\right)}^{b0}{\tilde{f}}^{b}-{\tilde{C}}_{b\left(t\right)}^{b0}\left(\nabla^{b}+w_{a}^{b}\right)=-C_{n0}^{b0}g^{n0}$$

Consequently, the vector observation model based on apparent gravity vectors for practical SINS is derived from Equation (14):

(15) $${\tilde{\beta }}_{a}+\delta \beta_{a}=-C_{n0}^{b0}\alpha_{a}$$

where

(16) $$\left\{\begin{array}{l} \begin{array}{c} {\tilde{\beta }}_{a}={\tilde{f}}^{b0}={\tilde{C}}_{b\left(t\right)}^{b0}{\tilde{f}}^{b}\\ \alpha_{a}=g^{n0}=C_{n\left(t\right)}^{n0}g^{n} \end{array}\\ \delta \beta_{a}=\left({\tilde{\phi }}^{b0}\times \right){\tilde{C}}_{b\left(t\right)}^{b0}{\tilde{f}}^{b}-{\tilde{C}}_{b\left(t\right)}^{b0}\left(\nabla^{b}+w_{a}^{b}\right) \end{array}\right.$$

As Equation (15) indicates, the apparent gravity observation vector incorporates multiple error terms. In addition to the random error term ${\tilde{C}}_{b\left(t\right)}^{b0}w_{a}^{b}$, there is also the constant error term ${\tilde{C}}_{b\left(t\right)}^{b0}\nabla^{b}$ and the variable error term $\left({\tilde{\phi }}^{b0}\times \right){\tilde{C}}_{b\left(t\right)}^{b0}{\tilde{f}}^{b}$. The magnitude of misalignment angle ${\tilde{\phi }}^{b0}$ correlates with both alignment duration and gyro bias magnitude. Evidently, the norm of the time-varying noise term $\left({\tilde{\phi }}^{b0}\times \right){\tilde{C}}_{b\left(t\right)}^{b0}{\tilde{f}}^{b}$ progressively increases with alignment time. This analysis demonstrates two critical aspects: First, the initial attitude result obtained by the coarse alignment method based on the inertial frame principle is a biased estimate, which corresponds to the limiting accuracy of coarse alignment. Second, the error of the biased estimation result calculated via the coarse alignment method also increases continuously. Consequently, to reduce the error drift of the alignment result, the alignment time should be shortened as much as possible.

### 2.4. Apparent Velocity Vector Observation Model

In the previous section, an apparent gravity vector observation model was constructed. As analyzed, the apparent gravity observation vector contains the random error term ${\tilde{C}}_{b\left(t\right)}^{b0}w_{a}^{b}$. However, the apparent gravity vector observation model only utilizes the vector information at the current moment, discarding all previous vector information. To reduce the influence of the random error term and fully utilize all vector information during the alignment process, a vector observation model based on apparent velocity will be derived and constructed below.

Integrating both sides of Equation (14) yields the vector observation model based on apparent velocity for solving initial attitude:

(17) $$\int_{0}^{t}{\tilde{C}}_{b\left(t\right)}^{b0}{\tilde{f}}^{b}dt+\int_{0}^{t}\left({\tilde{\phi }}^{b0}\times \right){\tilde{C}}_{b\left(t\right)}^{b0}{\tilde{f}}^{b}dt-\int_{0}^{t}{\tilde{C}}_{b\left(t\right)}^{b0}\left(\nabla^{b}+w_{a}^{b}\right)dt\;=-C_{n0}^{b0}\int_{0}^{t}C_{n\left(t\right)}^{n0}g^{n}dt$$

Assuming the accelerometer measurement noise $w_{a}^{b}$ follows a Gaussian distribution, the integral term $\int_{0}^{t}{\tilde{C}}_{b\left(t\right)}^{b0}w_{a}^{b}dt\approx 0$. Hence, Equation (17) becomes

(18) $$\int_{0}^{t}{\tilde{C}}_{b\left(t\right)}^{b0}{\tilde{f}}^{b}dt+\int_{0}^{t}\left({\tilde{\phi }}^{b0}\times \right){\tilde{C}}_{b\left(t\right)}^{b0}{\tilde{f}}^{b}dt-\int_{0}^{t}{\tilde{C}}_{b\left(t\right)}^{b0}\nabla^{b}dt=-C_{n0}^{b0}\int_{0}^{t}C_{n\left(t\right)}^{n0}g^{n}dt$$

Consequently, the observation vector and reference vector based on apparent velocity are extracted from Equation (18):

(19) $${\tilde{\beta }}_{v}+\delta \beta_{v}=-C_{n0}^{b0}\alpha_{v}$$

where

(20) $$\left\{\begin{array}{l} \begin{array}{l} {\tilde{\beta }}_{v}={\tilde{v}}^{b0}=\int_{0}^{t}{\tilde{C}}_{b\left(t\right)}^{b0}{\tilde{f}}^{b}dt\\ \alpha_{v}=v^{n0}=\int_{0}^{t}C_{n\left(t\right)}^{n0}g^{n}dt \end{array}\\ \delta \beta_{v}=\int_{0}^{t}\left({\tilde{\phi }}^{b0}\times \right){\tilde{C}}_{b\left(t\right)}^{b0}{\tilde{f}}^{b}dt-\int_{0}^{t}{\tilde{C}}_{b\left(t\right)}^{b0}\nabla^{b}dt \end{array}\right.$$

The discretization process is performed on Equation (20). Assume the current time is $t_{M}=M\cdot ∆t$, where M is a positive integer, and ∆t is the time interval of the SINS update period $\left(t_{k},\right.\;\left.t_{k+1}\right]$, with k=0,1,2,⋯,M−1 and $t_{k}=k\Delta t$. In Equation (20), the reference vector $\alpha_{v}$ can be calculated according to [35,36] as

(21) $$\begin{array}{ccc} \int_{0}^{t}C_{n\left(t\right)}^{n0}g^{n}dt & = & \sum_{k=0}^{M-1}\int_{t_{k}}^{t_{k+1}}C_{n\left(t\right)}^{n0}g^{n}dt=\sum_{k=0}^{M-1}C_{n\left(t_{k}\right)}^{n0}\int_{t_{k}}^{t_{k+1}}C_{n\left(t\right)}^{n\left(t_{k}\right)}g^{n}dt\\ & = & \sum_{k=0}^{M-1}C_{n\left(t_{k}\right)}^{n0}\left(∆tI_{3}+\frac{∆t^{2}}{2}\omega_{in}^{n}\times \right)g^{n} \end{array}$$

The observed vector ${\tilde{\beta }}_{v}$ in Equation (20) can be calculated according to [35,36] as

(22) $$\begin{array}{ccc} \int_{0}^{t}{\tilde{C}}_{b\left(t\right)}^{b0}{\tilde{f}}^{b}dt & = & \sum_{k=0}^{M-1}\int_{t_{k}}^{t_{k+1}}{\tilde{C}}_{b\left(t\right)}^{b0}{\tilde{f}}^{b}dt=\sum_{k=0}^{M-1}{\tilde{C}}_{b\left(t_{k}\right)}^{b0}\int_{t_{k}}^{t_{k+1}}{\tilde{C}}_{b\left(t\right)}^{b\left(t_{k}\right)}{\tilde{f}}^{b}dt\\ & \approx & \sum_{k=0}^{M-1}{\tilde{C}}_{b\left(t_{k}\right)}^{b0}\left(∆tI_{3}+\frac{∆t^{2}}{2}\omega_{ib}^{b}\times \right){\tilde{f}}^{b} \end{array}$$

Comparing Equation (18) with Equation (14) reveals that the apparent velocity vector eliminates the influence of accelerometer random errors on the initial alignment method through the integration of the apparent gravity vector. However, the accelerometer bias error term and the gyroscope bias error term remain. Moreover, due to the integration effect, the influence of the accelerometer and gyroscope bias error terms will become increasingly significant. Therefore, compared with the apparent gravity vector, the apparent velocity vector fully utilizes the vector information during the alignment process and can eliminate the influence of random errors on the system. Nevertheless, the integration also leads to a faster accumulation rate of vector drift errors. Ultimately, in terms of alignment error, the convergence process of the apparent velocity method is faster and smoother than that of the apparent gravity method, but it also exhibits a more rapid drift phenomenon.

### 2.5. Sliding Window Vector Integration Model

The preceding section noted that while the apparent velocity vector observation model can accelerate the convergence of coarse alignment, it accumulates inertial sensor errors in the observation vector. In this section, we employ the sliding-window vector integration method to effectively address this issue. The detailed process is as follows.

**Figure 1:**
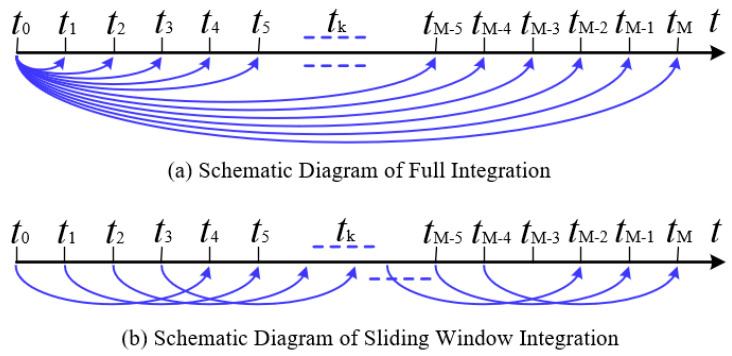
Schematic diagram of vector integration principle.

The schematic diagram of the vector integration principle is shown in Figure 1. It can be clearly observed from Figure 1 that when performing full integration on observation vectors, all previous observation vectors are involved in the calculation. This leads to the continuous accumulation of bias errors from gyroscope and accelerometer measurements within the observation vectors. In contrast, the sliding-window integration sampling algorithm only utilizes the information from the previous *S* sampling moments when solving for the current observation vector. Bias errors in gyroscope and accelerometer measurements from sampling moments outside the sliding window do not accumulate into the current observation vector. Therefore, the sliding-window vector integration method enhances the accuracy of observation vectors.

Assume that the starting time of the sliding window integration is $t_{s}$ and the current time is $t_{M}$. Therefore, the apparent velocity observation vector and reference vector based on sliding window vector integration can be extracted from Equation (19) as

(23) $${\tilde{\beta }}_{\Delta v}\left(t_{M}\right)+\delta \beta_{\Delta v}\left(t_{M}\right)=-C_{n0}^{b0}\alpha_{\Delta v}\left(t_{M}\right)$$

where

(24) $$\left\{\begin{array}{l} \begin{array}{l} {\tilde{\beta }}_{\Delta v}\left(t_{M}\right)=\int_{t_{S}}^{t_{M}}{\tilde{C}}_{b\left(t\right)}^{b0}{\tilde{f}}^{b}dt\\ \alpha_{\Delta v}\left(t_{M}\right)=\int_{t_{S}}^{t_{M}}C_{n\left(t\right)}^{n0}g^{n}dt \end{array}\\ \delta \beta_{\Delta v}\left(t_{M}\right)=\int_{t_{S}}^{t_{M}}\left({\tilde{\phi }}^{b0}\times \right){\tilde{C}}_{b\left(t\right)}^{b0}{\tilde{f}}^{b}dt-\int_{t_{S}}^{t_{M}}{\tilde{C}}_{b\left(t\right)}^{b0}\nabla^{b}dt \end{array}\right.$$

Analogous to the calculation method of full integration vectors, the discrete expressions of the reference vector and observation vector in Equation (24) are given as follows:

(25) $${\tilde{\beta }}_{\Delta v}\left(t_{M}\right)=\int_{t_{S}}^{t_{M}}{\tilde{C}}_{b\left(t\right)}^{b0}{\tilde{f}}^{b}dt\approx \sum_{k=S}^{M-1}{\tilde{C}}_{b\left(t_{k}\right)}^{b0}\left(∆tI_{3}+\frac{∆t^{2}}{2}\omega_{ib}^{b}\times \right){\tilde{f}}^{b}$$

(26) $$\alpha_{\Delta v}\left(t_{M}\right)=\int_{t_{S}}^{t_{M}}C_{n\left(t\right)}^{n0}g^{n}dt=\sum_{k=S}^{M-1}C_{n\left(t_{k}\right)}^{n0}\left(∆tI_{3}+\frac{∆t^{2}}{2}\omega_{in}^{n}\times \right)g^{n}$$

## 3. The Vector Reconstruction Model

In Equation (6), the apparent velocity observation vector model based on sliding window vector integration can be expressed as

(27) $$\beta_{\Delta v}=-C_{n0}^{b0}\int_{t_{S}}^{t}C_{n\left(t\right)}^{n0}g^{n}dt=-C_{n0}^{b0}\int_{t_{S}}^{t}C_{e0}^{n0}C_{e}^{e0}C_{n}^{e}g^{n}dt$$

According to [34], the expressions for matrices $C_{e0}^{n0}$, $C_{e}^{e0}$ and $C_{n}^{e}$ are given as

(28) $$C_{e0}^{n0}={\left(C_{n}^{e}\right)}^{T}=\left[\begin{array}{ccc} -\sin\lambda & \cos\lambda & 0\\ -\cos\lambda \sin L & -\sin\lambda \sin L & \cos L\\ \cos\lambda \cos L & \sin\lambda \cos L & \sin L \end{array}\right]$$

(29) $$C_{e}^{e0}=\left[\begin{array}{ccc} \cos\left(\omega_{ie}t\right) & -\sin\left(\omega_{ie}t\right) & 0\\ \sin\left(\omega_{ie}t\right) & \cos\left(\omega_{ie}t\right) & 0\\ 0 & 0 & 1 \end{array}\right]$$

where λ and L denote the longitude and latitude of the carrier, respectively. $\omega_{ie}$ is the Earth’s rotation rate, and the matrices $C_{n0}^{b0}$, $C_{e0}^{n0}$, and $C_{n}^{e}$ are constant. The matrix $C_{e}^{e0}$ is time-varying. Therefore, the apparent velocity observation vector model based on this can be expressed as

(30) $$\beta_{\Delta v}=K\Gamma \left(t\right)$$

where $K=C_{n0}^{b0}\phi_{\Delta v}$.

(31) $$\left\{\begin{array}{l} \phi_{\Delta v}=\left[\begin{array}{cccc} \frac{-g\cos L}{\omega_{ie}} & 0 & 0 & \frac{c\left(\omega_{ie}t_{S}\right)g\cos L}{\omega_{ie}}\\ 0 & \frac{-g\cos L\sin L}{\omega_{ie}} & g\cos L\sin L & \phi_{24}\\ 0 & \frac{g\;\cos^{2}L}{\omega_{ie}} & g\;\sin^{2}L & \phi_{34} \end{array}\right]\\ \Gamma \left(t\right)={\left[\begin{array}{ccc} \cos\left(\omega_{ie}t\right) & \sin\left(\omega_{ie}t\right) & \begin{array}{cc} t & 1 \end{array} \end{array}\right]}^{T} \end{array}\right.$$

where g denotes the local gravitational acceleration value. $\phi_{24}=-g\cos L\sin Lt_{S}+g\cos L\sin L\sin\left(\omega_{ie}t_{S}\right)/\omega_{ie}$, $\phi_{34}=-g\;\cos^{2}L\sin\left(\omega_{ie}t_{S}\right)/\omega_{ie}-g\;\sin^{2}Lt_{S}$. The complete derivation of Equation (31) appears in Appendix A.

Substituting Equation (30) into Equation (23) yields the following:

(32) $${\tilde{\beta }}_{\Delta v}=K\Gamma \left(t\right)+\delta ∆v^{b0}\left(t_{M}\right)$$

In the above equation, the matrix K is constant. The vector $\delta ∆v^{b0}\left(t_{M}\right)$ is the vector $\delta \beta_{\Delta v}\left(t_{M}\right)$ in Equation (24).

Assume that the three channels of the apparent velocity vector are non-coupled. Therefore, taking the first channel as an example and denoting it as x, we present a unified parameter identification model. For convenience, the ${\tilde{\beta }}_{\Delta v}\left(M\right)$ of the x channel is expressed as ${\tilde{\beta }}_{x,M}$.

(33) $${\tilde{\beta }}_{x,M}=K_{x,M}\Gamma \left(M\right)+\delta ∆v_{x,M}^{b0}$$

where $K_{x,M}$ represents the first row of the matrix $K_{M}$.

For the observation vector ${\tilde{\beta }}_{M}$ sampled at time $t_{M}$, analysis of the vector reconstruction model shows that the observation vector can be expressed as the sum of trigonometric functions and power functions, which can be decomposed into a power series form. In Equation (30), the true observation vector can be represented as a polynomial function of time. Here, let the dependent variable be the sampling time Δt, then the polynomial fitting model is established as follows:

(34) $${\tilde{\beta }}_{x,M}=\sum_{k=0}^{n}s_{x,k}{\Delta t}^{k}+\delta {\tilde{\beta }}_{x,M}=S_{x,M}T_{x,M}+\delta {\tilde{\beta }}_{x,M}$$

where the vectors $S_{x,M}$ and $T\left(M\right)$ are, respectively, as follows:

(35) $$S_{x,M}=\left[\begin{array}{ccc} s_{x,0} & s_{x,1} & \begin{array}{ccc} s_{x,2} & \ldots & s_{x,n} \end{array} \end{array}\right]$$

(36) $$T_{x,M}={\left[\begin{array}{ccc} 1 & \Delta t & \begin{array}{ccc} ∆t^{2} & \ldots & ∆t^{n} \end{array} \end{array}\right]}^{T}$$

Taking the matrix S as the observation vector and Equation (34) as the measurement equation, the filter model for Sage–Husa AKF is constructed as follows:

(37) $$\left\{\begin{array}{l} S_{x,M}=S_{x,M-1}\\ {\tilde{\beta }}_{x,M}=S_{x,M}T_{x,M}+V_{x,M} \end{array}\right.$$

where $V_{x,M}=\delta {\tilde{\beta }}_{x,M}=\delta ∆v_{x,M}^{b0}$. Assumedly, after polynomial fitting, only the random error term remains in the x channel of the observation vector, meaning $V_{x,M}$ follows a zero-mean Gaussian distribution, i.e., $E\left[V_{x,M}\right]=0$, $E\left[V_{x,M}{\left(V_{x,M}\right)}^{T}\right]=R_{x,M}$. In the filter model given by Equation (37), the state transition matrix is an identity matrix.

The Sage–Husa AKF method is implemented to estimate the fitting parameter $S_{x,M}$. As detailed in [37], the specific process is as follows:

(1) Calculate the measurement residual:

(38) $$e_{x,M}={\tilde{\beta }}_{x,M}-{\hat{S}}_{x,M}T_{x,M}$$

(2) The adaptive recursive estimation of the measurement noise covariance matrix:

(39) $${\hat{R}}_{x,M}=\left(1-d_{M}\right){\hat{R}}_{x,M-1}+d_{M}\left(e_{x,M}e_{x,M}^{T}-T_{x,M}^{T}P_{x,M}T_{x,M}\right)$$

(40) $$d_{M}=\frac{d_{M-1}}{d_{M-1}+b}$$

(3) Calculate the filter gain:

(41) $$K_{M}=P_{x,M}{T_{x,M}\left(T_{x,M}^{T}P_{x,M}T_{x,M}+{\hat{R}}_{x,M}\right)}^{-1}$$

(4) Update the state estimation:

(42) $${\hat{S}}_{x,M+1}={\hat{S}}_{x,M}+K_{M}e_{x,M}$$

(5) Update the mean square error of state estimation:

(43) $$P_{x,M+1}=P_{x,M}-K_{M}T_{x,M}^{T}P_{x,M}$$

where the initial value $d_{0}=1$. And 0<b<1 is termed the forgetting factor, typically selected within b=0.9~0.999.

After estimating the fitting parameter ${\hat{S}}_{x,M}$ using the AKF algorithm, the estimated parameter ${\hat{S}}_{x,M}$ is then used to recalculate the fitted apparent gravity vector as follows:

(44) $${\hat{\beta }}_{x,M}={\hat{S}}_{x,M}T_{x,M}$$

where ${\hat{\beta }}_{x,M}$ represents the fitted observation vector. Therefore, the new vector observation model can be obtained as

(45) $${\hat{\beta }}_{M}=C_{n0}^{b0}\alpha_{M}+\delta \beta_{M}$$

where $\delta \beta_{M}$ denotes the residual error after fitting the observation vector. Based on the aforementioned vector reconstruction method, the quaternion $q_{n0}^{b0}$ corresponding to the initial attitude matrix $C_{n0}^{b0}$ can be solved using the optimal-REQUEST attitude determination algorithm from a prior publication, thereby accomplishing the coarse alignment process. Specific steps of the attitude determination algorithm are detailed in Reference [30] and are not reiterated here.

## 4. Simulation Test

The effectiveness of the coarse alignment algorithm based on vector reconstruction via Sage–Husa AKF proposed in this paper is verified through simulation.

The parameters of the IMU in the simulation test are set as follows:

Gyroscopes biases: 0.02°/h;

Gyroscopes random noise: 0.005°/√h;

Accelerometers biases: 500 μg;

Accelerometers random noise: 50 μg/√Hz;

Output frequency: 200 Hz.

The simulation test will verify the performance of several coarse alignment methods through a swaying base, employing a sinusoidal motion model to simulate the swaying motion of the carrier under mooring or shaking conditions.

(46) $$\left\{\begin{array}{c} \theta =A_{\theta }\sin\left(2\pi f_{\theta }t+\phi_{\theta }\right)+\theta_{0}\\ \gamma =A_{\gamma }\sin\left(2\pi f_{\gamma }t+\phi_{\gamma }\right)+\gamma_{0}\\ \psi =A_{\psi }\sin\left(2\pi f_{\psi }t+\phi_{\psi }\right)+\psi_{0} \end{array}\right.$$

where parameters θ, γ and ψ represent the pitch, roll and yaw angle, parameters $A_{\theta }$, $A_{\gamma }$, and $A_{\psi }$ represent the swaying amplitudes; parameters $f_{\theta }$, $f_{\gamma }$, and $f_{\psi }$ represent the swaying frequencies; parameters $\phi_{\theta }$, $\phi_{\gamma }$, and $\phi_{\psi }$ represent the initial phases; and parameters $\theta_{0}$, $\gamma_{0}$, and $\psi_{0}$ represent the swaying centers. The swaying parameters in the simulation experiment are set as shown in Table 1. The alignment time is 200 s.

Linear vibration disturbances are introduced in the simulation test to evaluate algorithm performance. Under linear vibration excitation, the generated linear velocity vector of the carrier $V_{D}={\left[\begin{array}{ccc} V_{Dx} & V_{Dy} & V_{Dz} \end{array}\right]}^{T}$ is:

(47) $$\left\{\begin{array}{c} V_{Dx}=A_{Dx}\sin\left(\frac{2\pi }{T_{Dx}}t+\phi_{Dx}\right)\\ V_{Dy}=A_{Dy}\sin\left(\frac{2\pi }{T_{Dy}}t+\phi_{Dy}\right)\\ V_{Dz}=A_{Dz}\sin\left(\frac{2\pi }{T_{Dz}}t+\phi_{Dz}\right) \end{array}\right.$$

where, variables $A_{Dx}$, $A_{Dy}$ and $A_{Dz}$ represent the amplitude of disturbance linear velocity. Variables $T_{Dx}$, $T_{Dy}$ and $T_{Dz}$ represent the period of disturbance linear velocity. Variables $\phi_{Dx}$, $\phi_{Dy}$, and $\phi_{Dz}$ represent the initial phase of disturbance linear velocity. The amplitudes of disturbance linear velocity are 0.1 m/s, 0.1 m/s and 0.2 m/s, with periods of 6 s, 7 s, and 8 s respectively. The initial phase follows a uniform distribution in the interval [0, 2π].

**Figure 2:**
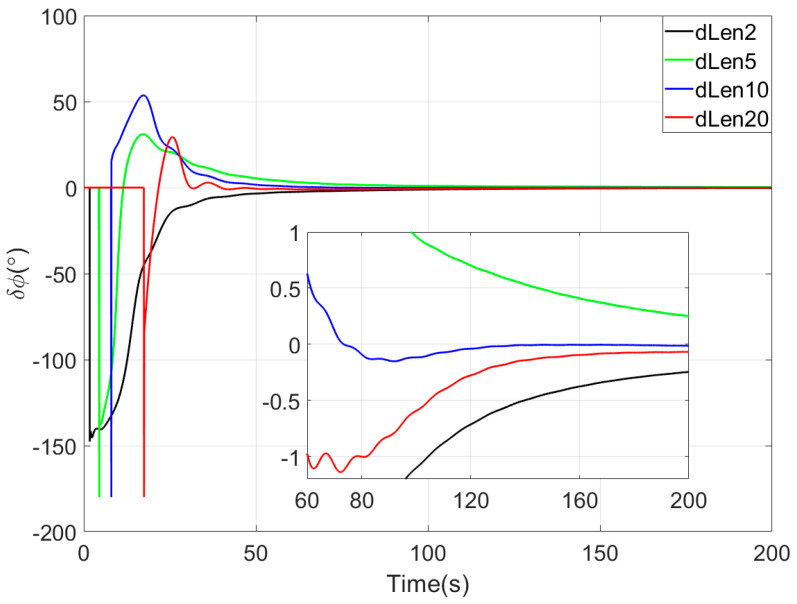
The yaw angle errors of the coarse alignment for four sliding-window lengths in simulation test.

In the sliding window vector integration method of the proposed coarse alignment algorithm, the sliding window length is a critical parameter. If the sliding window length is too short, the number of observation vectors involved in attitude determination is insufficient, leading to slow convergence of the coarse alignment. Conversely, if the window length is too long, an excessive number of observation vectors participate in attitude estimation, wherein the cumulative bias errors of inertial devices intensify, thereby degrading the accuracy of observation vector construction. In the simulation experiment, before comparing with other coarse alignment algorithms, the influence of different sliding window lengths on the performance of the proposed method was studied to select the optimal window length. In this experiment, four sliding window length parameters of 2 s, 5 s, 10 s, and 20 s were selected, named dLen2, dLen5, dLen10, and dLen20, respectively. For conciseness, this paper only details the comparison diagram of heading angle errors of the four sliding window length algorithms, as shown in Figure 2. The results show that the coarse alignment methods corresponding to the four sliding window length parameters can converge quickly. Comprehensive evaluation of convergence speed and alignment accuracy across the four algorithms, the coarse alignment algorithm with a sliding window length of 10 s has the best alignment performance. Therefore, in the subsequent simulation experiments and turntable tests, the sliding window length of the algorithm in this paper is uniformly set to 10 s.

Simultaneously, the initial filter parameters in Sage–Husa AKF are configured as follows: the state vector $S_{x}$ is a 4-dimensional vector, the forgetting factor b is set to 0.99, the initial measurement noise covariance matrix ${\hat{R}}_{x,\;0}$ is set to 0.1, and the initial state covariance matrix $P_{x,0}$ is set to ${1000I}_{4}$.

To thoroughly compare the performance of the algorithm proposed in this paper, three other coarse alignment algorithms are, respectively, introduced.

Scheme 1: the coarse alignment method with unprocessed observation vectors is denoted as Scheme 1, as described in [30].

Scheme 2: the coarse alignment method using a digital low-pass Infinite Impulse Response (IIR) filter for noise reduction processing of observation vectors is denoted as Scheme 2.

Scheme 3: the vector reconstruction coarse alignment algorithm using Kalman filter in [23,34] is denoted as Scheme 3.

Scheme 4: the vector reconstruction coarse alignment method based on the Sage–Husa AKF proposed in this paper is denoted as Scheme 4.

**Figure 3:**
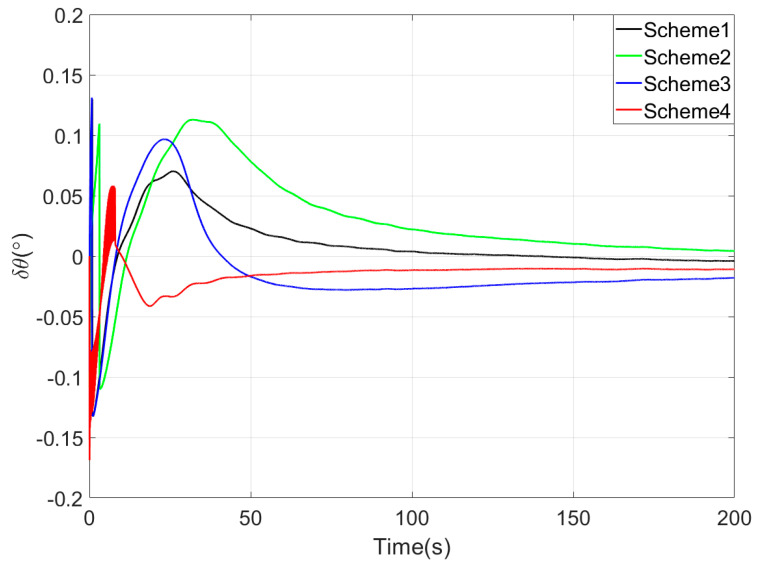
The pitch angle errors in simulation test.

**Figure 4:**
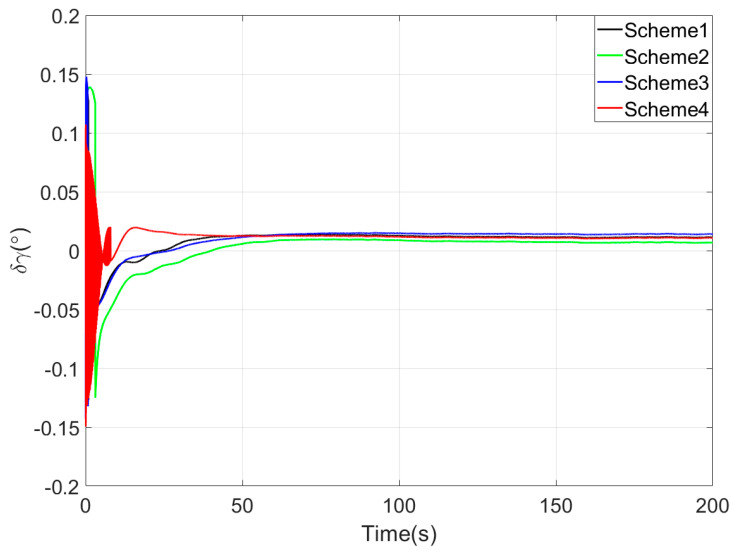
The roll angle errors in simulation test.

**Figure 5:**
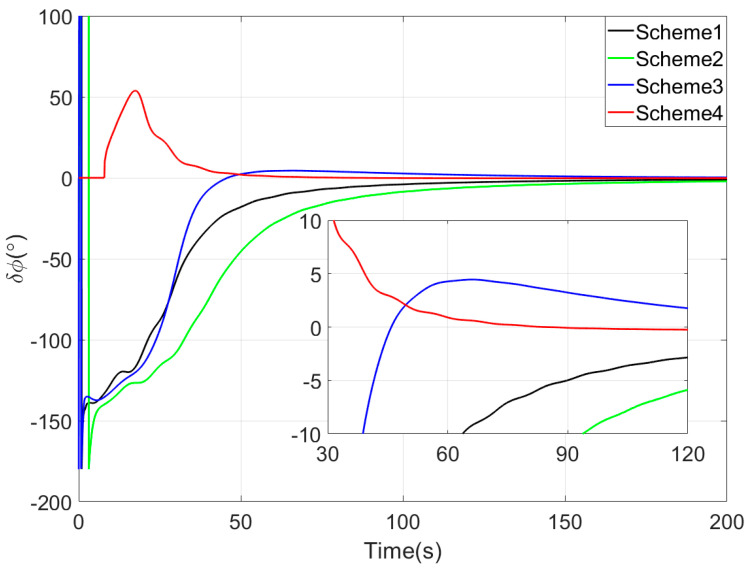
The yaw angle errors in simulation test.

Figure 3, Figure 4 and Figure 5 show the attitude error curves of the four coarse alignment algorithms under swaying conditions. The horizontal attitude error curves in Figure 3 and Figure 4 demonstrate that linear motion disturbances induce initial fluctuations in horizontal attitude errors across all four methods. Nevertheless, stable convergence is rapidly achieved in each case, with all horizontal attitude errors reaching within 0.02° by the end of the 200-s coarse alignment process. Comparatively, the proposed method exhibits significantly reduced susceptibility to such disturbances, attaining the fastest convergence rate for horizontal attitude errors.

Since the first 10 s fall within the sliding window length, the proposed coarse alignment algorithm did not output the heading angle. The heading angle error curve of Scheme 4 begins updating after 10 s. This is not a defect of the algorithm, and the method can also output the heading angle within the first 10 s.

Upon completion of the 200-s coarse alignment phase, heading angle errors for the four schemes are −1.0721°, −2.1035°, 0.2357°, and −0.1928° respectively. From both the final heading error results and the error curves in Figure 5, the following phenomena can be observed:

(1) All four coarse alignment methods are based on the apparent velocity vector observation model. Due to the integration effect, the apparent velocity vector effectively reduces the impact of random noise on the alignment results. Furthermore, all historical observation vectors are involved in the attitude determination. Consequently, the attitude error curves of all four methods converge smoothly.
(2) Both Scheme 3 and Scheme 4 can effectively process the observation vectors, and their attitude error curves show better convergence speed. However, due to the time-delay error introduced by the IIR filter, the alignment error curve of Scheme 2 has an obvious lag, performing worse than that of Scheme 1.
(3) Among the four coarse alignment algorithms, Scheme 4 has the best convergence speed and convergence accuracy performance. From the alignment results, the heading angle error of Scheme 4 can converge to within 1° in about 60 s.

The main reasons for the superior coarse alignment performance of the proposed algorithm are as follows. Affected by the bias errors of inertial sensors contained in observation vectors, the full integration algorithm causes continuous accumulation of bias errors in observation vectors, leading to slow drift of alignment errors. In contrast, the sliding window integration sufficiently utilizes vector information while avoiding error accumulation. Additionally, the vector reconstruction method based on the Sage–Husa AKF further improves the construction accuracy of observation vectors. Meanwhile, the optimal-REQUEST attitude determination algorithm realizes adaptive weight allocation of observation vectors by performing an optimal filter on measurement noise.

## 5. Turntable Test

To verify the performance of the proposed coarse alignment algorithm in practical systems, relevant validation tests were performed using a high-precision three-axis turntable.

**Figure 6:**
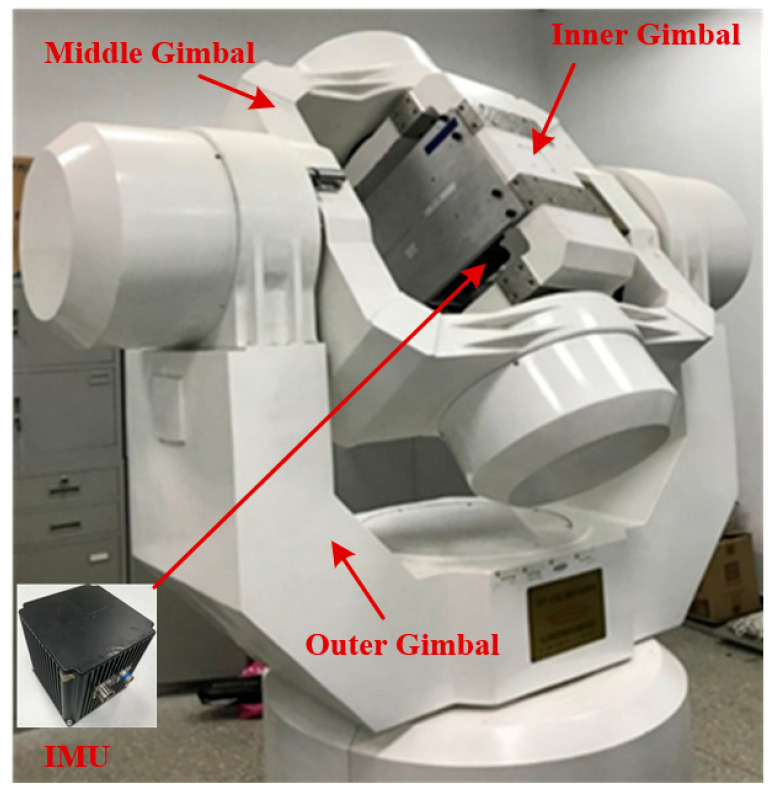
The physical diagram of turntable.

**Figure 7:**
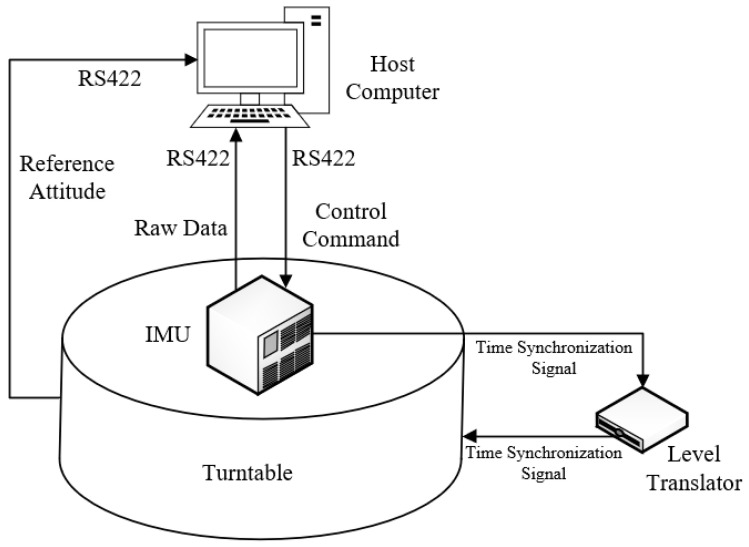
The structural schematic diagram of the turntable test.

The inner frame, middle frame, and outer frame of the three-axis multi-functional turntable correspond to the roll angle, pitch angle, and heading angle of the SINS, respectively. The parameters of the IMU used in the turntable test are shown in Table 2. The physical diagram of the three-axis multi-functional turntable is shown in Figure 6, and the structural schematic diagram of the turntable test is shown in Figure 7. Before the turntable test, the IMU was calibrated to compensate for the scale factor errors, installation errors, bias errors, etc., of the gyroscopes and accelerometers. As shown in Figure 7, the data acquisition system simultaneously acquires IMU data and turntable attitude data via synchronous signals. After calibrating the installation errors between the IMU and turntable, the real-time turntable data can serve as the attitude reference for the turntable test.

The experimental scheme for coarse alignment on a swaying base is as follows: The IMU is installed at the center of the three-axis turntable, and installation errors and inertial sensors’ bias errors are calibrated in advance. The motion scheme of three-axis sinusoidal sway is designed for turntable tests under swaying conditions. The motion settings of the three-axis turntable under swaying conditions are as follows: the swaying center of the inner frame (roll angle) of the turntable is 0°, the amplitude is 5°, and the frequency is 0.15 Hz. The swaying center of the middle frame (pitch angle) is 0°, the amplitude is 4°, and the frequency is 0.125 Hz. The swaying center of the outer frame (yaw angle) is 0°, the amplitude is 3°, and the frequency is 0.2 Hz. The coarse alignment test time is set to 200 s.

As in the simulation experiments, three additional coarse alignment algorithms were introduced to conduct a comparative performance analysis with the proposed algorithm.

In the turntable test, the four comparative methods (Scheme 1~Scheme 4) follow the same definition approaches as in the simulation test. And the initial parameters of the filter model for the turntable test are configured identically to those in the simulation test.

**Figure 8:**
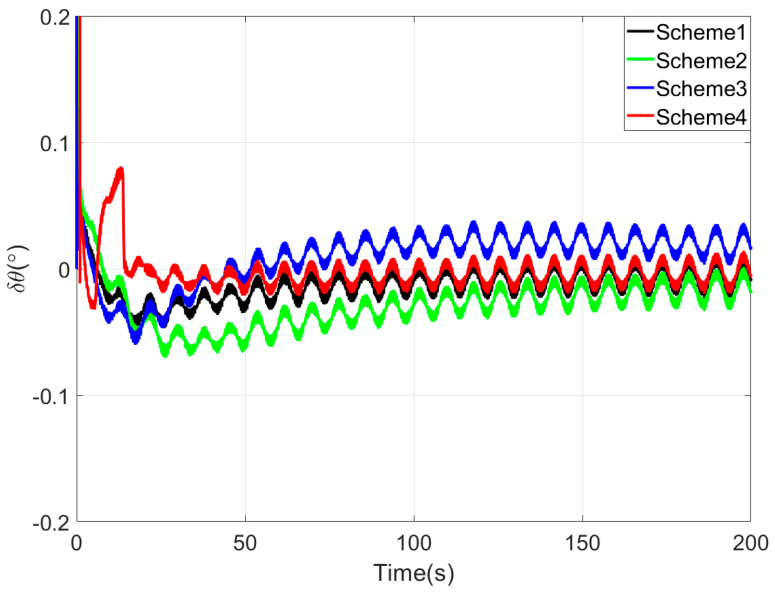
The pitch angle errors in turntable test.

**Figure 9:**
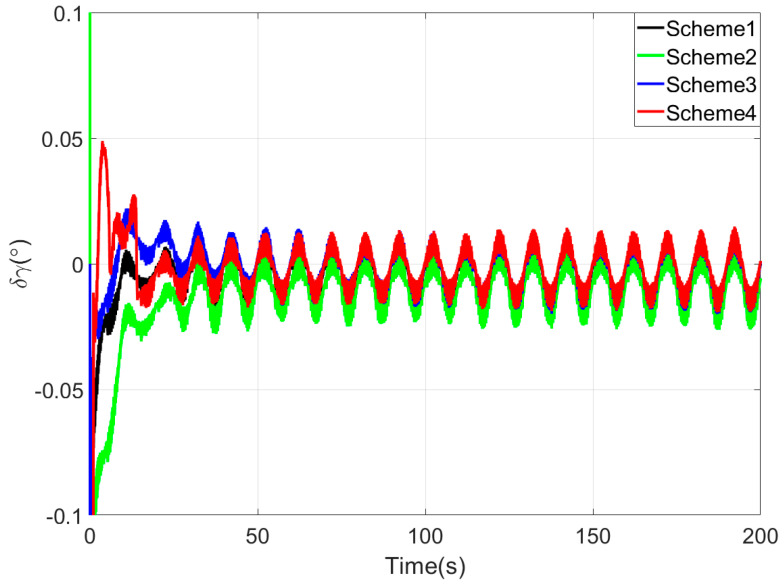
The roll angle errors in turntable test.

**Figure 10:**
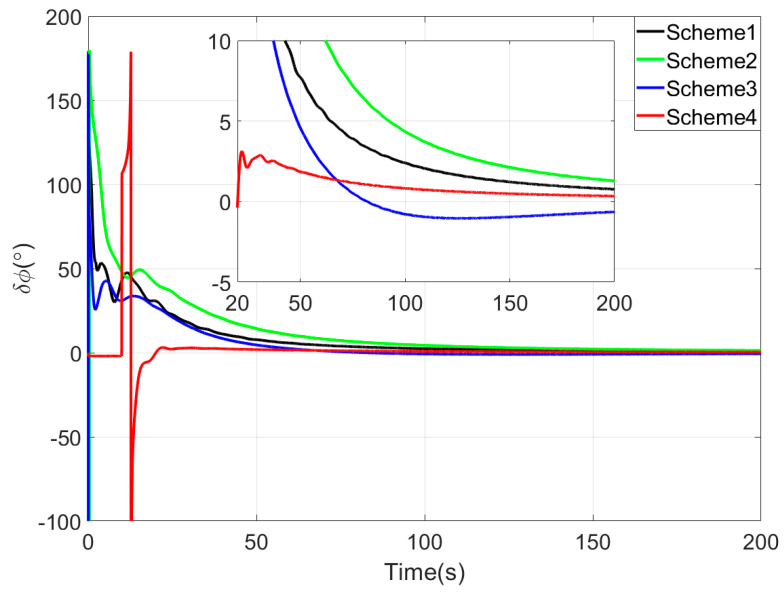
The yaw angle errors in turntable test.

Figure 8, Figure 9 and Figure 10 present the comparison curves of pitch angle error, roll angle error, and heading angle error of the four coarse alignment algorithms under swaying conditions. As shown in Figure 8 and Figure 9, the horizontal angle errors of the four coarse alignment methods can reach the limiting alignment accuracy within a short time.

Since the first 10 s fall within the sliding window length, the proposed coarse alignment algorithm did not output the heading angle. The heading angle error curve of Scheme 4 begins updating after 10 s. By comparing the heading angle error curve in Figure 10, the following results can be obtained.

Similarly to the simulation experiment, due to the delay error of the IIR filter, the alignment error curve of Scheme 2 has an obvious lag, so the performance of Scheme 2 is worse than that of Scheme 1.

Among the four coarse alignment algorithms, Scheme 4 demonstrates the optimal performance in both convergence speed and accuracy.

To compare the performance of the four coarse alignment algorithms under swaying conditions more clearly and specifically, the mean error (MEAN), standard deviation (STD), and root mean square error (RMSE) of the attitude errors for each method were statistically analyzed. Equations (48)–(51) provides the mathematical formulas for the statistical errors of MEAN, STD, and RMSE. To compare the convergence speed and accuracy of the four coarse alignment methods, respectively, the attitude errors were statistically analyzed in 20~50 s and 100~200 s intervals. Table 3 presents the error statistics of the alignment errors during the turntable test for the four coarse alignment algorithms.

(48) $$\varepsilon_{MEAN}=\frac{1}{n}\sum_{i=1}^{n}\left({\tilde{x}}_{i}-x_{i}\right)$$

(49) $$\bar{x}=\frac{1}{n}\sum_{i=1}^{n}{\tilde{x}}_{i}$$

(50) $$\varepsilon_{STD}=\sqrt{\frac{1}{n-1}\sum_{i=1}^{n}{\left({\tilde{x}}_{i}-\bar{x}\right)}^{2}}$$

(51) $$\varepsilon_{RMSE}=\sqrt{\frac{1}{n}\sum_{i=1}^{n}{\left({\tilde{x}}_{i}-x_{i}\right)}^{2}}$$

where, $\varepsilon_{MEAN}$ represents the mean error, ${\tilde{x}}_{i}$ represents the *i*-th calculated angle, $x_{i}$ represents the *i*-th reference angle, n represents the number of calculated angles, $\bar{x}$ represents the average value of calculated angles, $\varepsilon_{STD}$ represents the standard deviation error, $\varepsilon_{RMSE}$ represents the root mean square error.

As shown in the table, the statistical results of horizontal attitude angle errors of the four coarse alignment methods are close. Based on the statistical results of attitude errors at 20~50 s intervals, the proposed Scheme 4 method exhibits the lowest heading angle error among the four methods, indicating the fastest convergence speed. According to 100~200 s attitude error statistics, Scheme 4 achieves the most minor heading angle error, demonstrating superior convergence accuracy.

Based on a comprehensive analysis, the proposed coarse alignment method achieves horizontal attitude errors better than 0.01° within 20 s and maintains heading errors within ±0.6° after 100 s under swaying conditions. Turntable test results demonstrate that the proposed method delivers superior alignment performance compared to the other three coarse alignment algorithms.

## 6. Conclusions

In this paper, a coarse alignment method based on vector reconstruction via Sage–Husa AKF on a swaying base is proposed. The accumulation of inertial device errors and external disturbances degrades the accuracy of the coarse alignment observation vector, thereby limiting self-alignment performance. In response to this issue, this paper proposes three improvements. Firstly, a sliding-window vector integration method is proposed to enhance the construction accuracy of the apparent velocity observation vector, effectively preventing continuous accumulation of inertial instrument errors while suppressing random noise. A vector reconstruction model based on the Sage–Husa AKF algorithm is proposed, significantly reducing the impact of external disturbances on observation vector accuracy. The proposed optimal-REQUEST attitude determination method effectively enhances coarse alignment performance by adaptively adjusting observation vector weights. Simulations and turntable experiments were conducted to validate the performance of the coarse alignment algorithm proposed in this paper. The results demonstrate that the proposed method significantly enhances convergence speed and accuracy of coarse alignment. Meanwhile, the proposed method can also be applied to the in-motion integrated alignment methods to enhance their performance, which constitutes a subsequent research plan of our team.

## Figures and Tables

**Table 1:** The parameter settings for the swaying motion in the simulation test.

Parameters	Pitch	Roll	Yaw
amplitude $\left({}^{\circ}\right)$	8	10	6
frequency $\left(Hz\right)$	0.15	0.125	0.2
initial phase $\left({}^{\circ}\right)$	0	0	0
swaying center $\left({}^{\circ}\right)$	0	0	0

**Table 2:** The parameters of IMU in turntable test.

Sensors	Parameters	Value
Gyroscope	Constant biases	≤0.01°/h (1σ)
	Random noise	≤0.005°/√h
	Measurement range	±300°/s
	Output frequency	200 Hz
Accelerometer	Constant biases	$\leq 5\times 10^{-4}g$
	Random noise	$\leq 5\times 10^{-4}\;g$
	Measurement range	±20 g
	Output frequency	200 Hz

**Table 3:** The statistical results of attitude error in turntable test.

Errors		20~50 s				100~200 s			
		Scheme 1	Scheme 2	Scheme 3	Scheme 4	Scheme 1	Scheme 2	Scheme 3	Scheme 4
Pitch angle Error (°)	MEAN	−0.0276	−0.0537	−0.0177	0.0002	−0.0075	−0.0206	0.0217	−0.0001
	STD	0.0070	0.0080	0.0135	0.0064	0.0079	0.0087	0.0078	0.0078
	RMS	0.0285	0.0543	0.0223	0.0064	0.0109	0.0223	0.023	0.0078
Roll angle Error (°)	MEAN	−0.0028	−0.0127	0.0024	−0.0039	−0.0030	−0.0091	−0.0023	−0.0037
	STD	0.0062	0.0066	0.0069	0.0063	0.0078	0.0078	0.0078	0.0078
	RMS	0.0068	0.0143	0.0073	0.0074	0.0084	0.0120	0.0082	0.0087
Yaw angle Error (°)	MEAN	15.4765	25.6034	13.0368	2.3635	1.3306	2.3394	−0.8883	0.5193
	STD	6.3201	8.0633	6.6475	0.4128	0.4460	0.8402	0.1221	0.1337
	RMS	16.7170	26.8428	14.6335	2.3992	1.4033	2.4857	0.8966	0.5362

## Data Availability

The data presented in this study are available on request from the corresponding author. The data are not publicly available due to the privacy of the subjects involved in the study.

## Appendix A

This section primarily presents the detailed derivation process of Equation (31). For notational convenience, the sine function $\sin\left(\cdot \right)$ is abbreviated as $s\left(\cdot \right)$, and the cosine function $\cos\left(\cdot \right)$ as $c\left(\cdot \right)$. As can be seen from Equations (27)–(29), the specific expression of vector $\beta_{\Delta v}$ is given by:

(A1) $$\beta_{\Delta v}=-C_{n0}^{b0}\int_{t_{S}}^{t}C_{e0}^{n0}C_{e}^{e0}C_{n}^{e}g^{n}dt$$

Based on the associative property of matrix multiplication ABC = A(BC), the expression $-C_{e}^{e0}C_{n}^{e}g^{n}$ is first derived, with the detailed procedure presented below:

(A2) $$\begin{array}{c} \left[\begin{array}{ccc} c\left(\omega_{ie}t\right) & -s\left(\omega_{ie}t\right) & 0\\ s\left(\omega_{ie}t\right) & c\left(\omega_{ie}t\right) & 0\\ 0 & 0 & 1 \end{array}\right]\left[\begin{array}{ccc} -s\lambda & -c\lambda sL & c\lambda cL\\ c\lambda & -s\lambda sL & s\lambda cL\\ 0 & cL & sL \end{array}\right]\left[\begin{array}{c} 0\\ 0\\ g \end{array}\right]\\ \;\;\;\;\;\;=\left[\begin{array}{ccc} c\left(\omega_{ie}t\right) & -s\left(\omega_{ie}t\right) & 0\\ s\left(\omega_{ie}t\right) & c\left(\omega_{ie}t\right) & 0\\ 0 & 0 & 1 \end{array}\right]\left[\begin{array}{c} gc\lambda cL\\ gs\lambda cL\\ gsL \end{array}\right]\\ \;\;\;\;\;\;=\left[\begin{array}{c} c\left(\omega_{ie}t\right)gc\lambda cL-s\left(\omega_{ie}t\right)gs\lambda cL\\ s\left(\omega_{ie}t\right)gc\lambda cL+c\left(\omega_{ie}t\right)gs\lambda cL\\ gsL \end{array}\right]\\ \;\;\;\;\;\;=\left[\begin{array}{ccc} gc\lambda cL & -gs\lambda cL & 0\\ gs\lambda cL & gc\lambda cL & 0\\ 0 & 0 & gsL \end{array}\right]\left[\begin{array}{c} c\left(\omega_{ie}t\right)\\ s\left(\omega_{ie}t\right)\\ 1 \end{array}\right] \end{array}$$

Substitution of Equation (A2) into Equation (A1) yields:

(A3) $$\begin{array}{c} \beta_{\Delta v}=C_{n0}^{b0}\int_{t_{S}}^{t}\left[\begin{array}{ccc} -s\lambda & c\lambda & 0\\ -c\lambda sL & -s\lambda sL & cL\\ c\lambda cL & s\lambda cL & sL \end{array}\right]\left[\begin{array}{ccc} gc\lambda cL & -gs\lambda cL & 0\\ gs\lambda cL & gc\lambda cL & 0\\ 0 & 0 & gsL \end{array}\right]\left[\begin{array}{c} c\left(\omega_{ie}t\right)\\ s\left(\omega_{ie}t\right)\\ 1 \end{array}\right]dt\\ =C_{n0}^{b0}\left[\begin{array}{ccc} -s\lambda & c\lambda & 0\\ -c\lambda sL & -s\lambda sL & cL\\ c\lambda cL & s\lambda cL & sL \end{array}\right]\left[\begin{array}{ccc} gc\lambda cL & -gs\lambda cL & 0\\ gs\lambda cL & gc\lambda cL & 0\\ 0 & 0 & gsL \end{array}\right]\int_{t_{S}}^{t}\left[\begin{array}{c} c\left(\omega_{ie}t\right)\\ s\left(\omega_{ie}t\right)\\ 1 \end{array}\right]dt \end{array}$$

The above integral expression is first derived separately.

(A4) $$\begin{array}{cc} \int_{t_{S}}^{t}\left[\begin{array}{c} c\left(\omega_{ie}t\right)\\ s\left(\omega_{ie}t\right)\\ 1 \end{array}\right]dt= & {\left.\begin{array}{c} \frac{s\left(\omega_{ie}t\right)}{\omega_{ie}}\\ \frac{-c\left(\omega_{ie}t\right)}{\omega_{ie}}\\ t \end{array}\right|}_{t_{S}}^{t}=\left[\begin{array}{c} \frac{s\left(\omega_{ie}t\right)-s\left(\omega_{ie}t_{S}\right)}{\omega_{ie}}\\ \frac{-c\left(\omega_{ie}t\right)+c\left(\omega_{ie}t_{S}\right)}{\omega_{ie}}\\ t-t_{S} \end{array}\right]\\ & =\left[\begin{array}{cccc} 0 & \frac{1}{\omega_{ie}} & 0 & \frac{-s\left(\omega_{ie}t_{S}\right)}{\omega_{ie}}\\ \frac{-1}{\omega_{ie}} & 0 & 0 & \frac{c\left(\omega_{ie}t_{S}\right)}{\omega_{ie}}\\ 0 & 0 & 1 & -t_{S} \end{array}\right]\left[\begin{array}{c} c\left(\omega_{ie}t\right)\\ \begin{array}{c} s\left(\omega_{ie}t\right)\\ t\\ 1 \end{array} \end{array}\right] \end{array}$$

Substitution of Equation (A4) into Equation (A3) yields:

(A5) $$\begin{array}{c} \beta_{\Delta v}=C_{n0}^{b0}\left[\begin{array}{ccc} -s\lambda & c\lambda & 0\\ -c\lambda sL & -s\lambda sL & cL\\ c\lambda cL & s\lambda cL & sL \end{array}\right]\left[\begin{array}{ccc} gc\lambda cL & -gs\lambda cL & 0\\ gs\lambda cL & gc\lambda cL & 0\\ 0 & 0 & gsL \end{array}\right]\left[\begin{array}{cccc} 0 & \frac{1}{\omega_{ie}} & 0 & \frac{-s\left(\omega_{ie}t_{S}\right)}{\omega_{ie}}\\ \frac{-1}{\omega_{ie}} & 0 & 0 & \frac{c\left(\omega_{ie}t_{S}\right)}{\omega_{ie}}\\ 0 & 0 & 1 & -t_{S} \end{array}\right]\left[\begin{array}{c} c\left(\omega_{ie}t\right)\\ \begin{array}{c} s\left(\omega_{ie}t\right)\\ t\\ 1 \end{array} \end{array}\right]\\ \;\;\;\;\;\;=C_{n0}^{b0}\left[\begin{array}{ccc} -s\lambda & c\lambda & 0\\ -c\lambda sL & -s\lambda sL & cL\\ c\lambda cL & s\lambda cL & sL \end{array}\right]\left[\begin{array}{cccc} \frac{gs\lambda cL}{\omega_{ie}} & \frac{gc\lambda cL}{\omega_{ie}} & 0 & \mu_{14}\\ \frac{-gc\lambda cL}{\omega_{ie}} & \frac{gs\lambda cL}{\omega_{ie}} & 0 & \mu_{24}\\ 0 & 0 & gsL & -t_{S}gsL \end{array}\right]\left[\begin{array}{c} c\left(\omega_{ie}t\right)\\ \begin{array}{c} s\left(\omega_{ie}t\right)\\ t\\ 1 \end{array} \end{array}\right]\\ \;\;\;\;\;\;=C_{n0}^{b0}\left[\begin{array}{cccc} \frac{-gcL}{\omega_{ie}} & 0 & 0 & \frac{c\left(\omega_{ie}t_{S}\right)gcL}{\omega_{ie}}\\ 0 & \frac{-gcLsL}{\omega_{ie}} & gcLsL & \phi_{24}\\ 0 & \frac{gc^{2}L}{\omega_{ie}} & gs^{2}L & \phi_{34} \end{array}\right]\left[\begin{array}{c} c\left(\omega_{ie}t\right)\\ \begin{array}{c} s\left(\omega_{ie}t\right)\\ t\\ 1 \end{array} \end{array}\right]=C_{n0}^{b0}\phi_{\Delta v}\Gamma \left(t\right) \end{array}$$

where,

(A6) $$\mu_{14}=\left(-s\left(\omega_{ie}t_{S}\right)gc\lambda cL-c\left(\omega_{ie}t_{S}\right)gs\lambda cL\right)/\omega_{ie}$$

(A7) $$\mu_{24}=\left(-s\left(\omega_{ie}t_{S}\right)gs\lambda cL+c\left(\omega_{ie}t_{S}\right)gc\lambda cL\right)/\omega_{ie}$$

(A8) $$\phi_{24}=-gcLsLt_{S}+gcLsLs\left(\omega_{ie}t_{S}\right)/\omega_{ie}$$

(A9) $$\phi_{34}=-gc^{2}Ls\left(\omega_{ie}t_{S}\right)/\omega_{ie}-gs^{2}Lt_{S}$$
